# Prevalence of allergen sensitization among children with allergic rhinitis in Changzhou, China: a retrospective observational study

**DOI:** 10.1186/s12887-023-04291-9

**Published:** 2023-09-16

**Authors:** Zhibang Hu, Jianrong Xue, Min Pan, Yongzheng Bao, Wenlan Zou, Chunhui Wang, Jing Ma

**Affiliations:** 1 Department of Otorhinolaryngology, the Third People’s Hospital of Changzhou, Changzhou, 213001 China; 2 Department of Pharmacy, the Third People’s Hospital of Changzhou, Changzhou, 213001 China

**Keywords:** Allergen, Children, Prevalence, Specific IgE, Changzhou, China

## Abstract

**Objective:**

To determine the prevalence of sensitivity to common inhaled and food allergens among children with allergic rhinitis (AR) in Changzhou in eastern China and provide a basis for epidemiological research of pediatric allergic rhinitis and allergen avoidance in this region.

**Methods:**

This was a retrospective observational study, a total of 1248 children with AR were enrolled at the Third People’s Hospital of Changzhou between January 2018 and December 2019. The serum-specific immunoglobulin E (sIgE) to 19 kinds of inhaled and food allergens and serum total IgE were detected with the AllergyScreen test (Mediwiss Analytic GmbH, Moers, Germany). All participants had a positive reaction to at least one allergen in the test (the sIgE concentration ≥ 0.35 IU/ml).

**Results:**

Among the patients, 818 (65.54%) were male and 430 (34.46%) were female, with 81 (6.50%) aged 1–3 year, 501 (40.14%) aged 4–7 year, and 666 (53.36%) aged 8–14 year. The positivity rate of inhaled allergens was 80.05% (*n* = 999), while the positivity rate of food allergens was 66.19% (*n* = 826). 828 patients (66.35%) were sensitized to multiple allergens. The most common inhaled allergens were *Dermatophagoides pteronyssinus* (65.38%), mold mix (25.56%), house dust (20.67%), and dog hair dander (13.94%), and the most common food allergens were cow’s milk (30.31%), cashew nut (27.9%), egg (22.68%), and beef (12.98%). With an increase in age, the inhaled allergen positivity rate showed a significant increase (*P* < 0.01), while the food allergen positivity rate decreased significantly (*P* < 0.01). There were significant age differences in total IgE levels (*P* < 0.01) and the total IgE level was highest in the group aged 8–14 year.

**Conclusions:**

*Dermatophagoides pteronyssinus* was the most common sensitizing allergen in pediatric patients with AR in Changzhou. Several other inhaled and food allergens were also common. We observed that multiple allergenic factors play an important role in the occurrence and development of AR.

Allergic rhinitis (AR) is a non-infectious, chronic inflammatory disease of the nose mediated by IgE after exposure to allergens in atopic individuals of all ages, especially children and adolescents [1, 2]. The worldwide prevalence rate of AR is 10–40% and it affects more than 20% of the Chinese population, a trend that is increasing yearly [3–5]. Because it influences the occurrence, development, and treatment outcomes of lower respiratory tract inflammatory diseases, especially bronchial asthma, AR seriously reduces quality of life in children, increases the family medical expenditure, and directly or indirectly causes serious medical consequences and social burden [3]. Pediatric AR treatment mainly includes environmental control, medications, and immunotherapy [6]. The primary goal of AR treatment in children is to improve their living environment by reducing allergen exposure, especially as an early intervention for infants [7]. These reduction measures can help to reduce the production of specific IgE and may even eradicate the allergen sensitization state. Clinically, children with AR present with multiple sensitization factors [8]. Analysis of the distribution characteristics of allergens, especially those that are specific to AR, can provide an important reference for the diagnosis and treatment of this disease [9]. In this study, we analyzed the allergen sensitivity status in children with AR in Changzhou, eastern China, to facilitate the prevention, diagnosis, and management of AR in this region.

## Methods

### Study design

In this retrospective study, a total of 1248 consecutive pediatric patients diagnosed with AR by in vitro assay of specific IgE (sIgE) to common allergens were enrolled from January 2018 to December 2019 in the Third People’s Hospital of Changzhou, Jiangsu Province, China. All children with AR were in compliance with the Chinese guidelines for the diagnosis and treatment of AR in children [4, 10]. All participating children had tested positive for sIgE to at least one of the 19 allergens (the sIgE concentration ≥ 0.35 IU/ml).

### AllergyScreen test

A total of 19 inhaled and food allergen-specific lgE (sIgE) and total serum IgE levels were measured in serum using the AllergyScreen test (Mediwiss Analytic GmbH, Moers, Germany) and its specialized in vitro allergen diagnostic reagents according to the manufacturer’s protocol. Inhaled allergens included *Dermatophagoides pteronyssinus*, house dust, mulberry, cat dander, dog hair, cockroach, amaranth, mold mix, grass mix, and tree pollen mix. The food allergens included egg, cow’s milk, shrimp, beef, shellfish, crab, mango, cashew nut, and pineapple. The results were semi-quantified and classified with the following grading criteria: grade 0 (< 0.35 IU/ml); grade I (0.35–0.70 IU/ml); grade II (0.71–3.5 IU/ml); grade III (3.6–17.5 IU/ml); grade IV (17.6–50 IU/ml); grade V (51–100 IU/ml); and grade VI (> 100 IU/ml). A sIgE concentration ≥ 0.35 IU/ml was considered positive. The total serum IgE level was divided into three groups: the low-concentration group (< 100 IU/ml); the medium-concentration group (100–200 IU/ml); and the high-concentration group (> 200 IU/ml).

### Sample size

Our center was the largest diagnosis and treatment center for allergic rhinitis in Changzhou, and the number of patients in our center during the study period determined the sample size.

### Statistical analysis

All data were analyzed using the IBM SPSS Statistics version 21. The sIgE positivity rates were compared with the chi-squared test or Fisher’s exact probability method and total IgE levels were compared with the Kruskal-Wallis test and the Mann-Whitney U test. *P* < 0.05 was considered statistically significant.

## Results

### Overall positivity rate of sIgE with inhaled and food allergens

420 patients (33.65%) were sensitized to only one allergen, and 828 patients (66.35%) were sensitized to multiple allergens. The positivity rate of serum inhaled allergens in children with AR was 999(80.05%), significantly higher than the food allergen positivity rate of 826(66.19%) (*P* < 0.01). The most common inhaled allergens were *D. pteronyssinus* 816 (65.38%), mold mix 319 (25.56%), house dust 258 (20.67%), and dog hair 174 (13.94%). Similarly, among the food allergens, positivity rates of sIgE to cow’s milk 377 (30.21%), cashew nut 349 (27.9%), egg 283 (22.68%), and beef 162 (12.98%) were more frequent (Table 1).

**Table 1:** The positivity rate of sIgE with inhaled and food allergens

Allergens		Negative (Grade 0)	Positive							
			Grade I	Grade II	Grade III	Grade IV	Grade V	Grade VI	Total	Ranking
Inhaled	D. *pteronyssinus*	432 (34.62%)	33	95	234	225	105	124	816 (65.38%)	1
	House dust	990 (79.33%)	105	141	9	2	0	1	258 (20.67%)	6
	Mulberry	1215 (97.36%)	12	18	2	1	0	0	33 (2.64%)	17
	Cat dander	1107 (88.70%)	20	65	46	7	0	3	141 (11.30%)	10
	Dog hair	1074 (86.06%)	82	86	4	2	0	0	174 (13.94%)	7
	Cockroach	1201 (96.23%)	27	17	2	0	1	0	47 (3.77%)	15
	Amaranth	1168 (93.59%)	29	26	20	5	0	0	80 (6.41%)	13
	Mold mix	929 (74.44%)	46	95	134	36	7	1	319 (25.56%)	4
	Grass mix	1217 (97.52%)	13	11	6	1	0	0	31 (2.48%)	18
	Tree pollen mix	1175 (94.15%)	34	32	5	1	1	0	73 (5.85%)	14
Food	Egg	965 (77.32%)	148	115	15	1	4	0	283 (22.68%)	5
	Cow’s milk	871 (69.79%)	152	210	13	1	0	1	377 (30.21%)	2
	Shrimp	1136 (91.03%)	56	29	20	3	0	4	112 (8.97%)	11
	Beef	1086 (87.02%)	120	42	0	0	0	0	162 (12.98%)	8
	Shellfish	1203 (96.39%)	41	3	1	0	0	0	45 (3.61%)	16
	Crab	1092 (87.50%)	70	46	26	8	1	5	156 (12.50%)	9
	Mango	1161 (93.03%)	68	16	3	0	0	0	87 (6.97%)	12
	Cashew nut	899 (72.04%)	163	132	41	13	0	0	349 (27.96%)	3
	Pineapple	1224 (98.08%)	16	8	0	0	0	0	24 (1.92%)	19

### Allergen sensitization in different sex and age groups

The positivity rates of overall inhaled allergens in both boys (81.05%, 663/818) and girls (78.14%, 336/430) were significantly higher than the positivity rates of food allergens in boys (65.77%, 538/818) and girls (66.98%, 288/430) (*P* < 0.01) (Table 2). There were no sex differences in the overall positive rates of inhaled (81.05% vs. 78.14%) and food allergens (65.77% vs. 66.98%) (*P* > 0.05) (Table 2). However, the sIgE positive rates of mulberry, cat dander, dog hair, amaranth, beef, and crab were higher among males than females (*P* < 0.05) (Table 3).

**Table 2:** The positivity rates of overall inhaled and food allergens compared by sex

Allergens	Inhaled	Food	*P* value
Male	663 (81.05%)	538 (65.77%)	< 0.001
Female	336 (78.14%)	288 (66.98%)	< 0.001
*P* value	0.22	0.67	

**Table 3:** Allergen sensitization compared by sex

Allergens	Number of sIgE-positive patients (%)		*P* value
	Male	Female	
*D. pteronyssinus*	542(66.26%)	274(63.72%)	0.37
House dust	169(20.66%)	89(20.70%)	0.99
Mulberry	29(3.55%)	4(0.93%)	0.01
Cat dander	105(12.84%)	36(8.37%)	0.02
Dog hair	130(15.89%)	44(10.23%)	0.01
Cockroach	33(4.03%)	14(3.26%)	0.49
Amaranth	64(7.82%)	16(3.72%)	0.01
Mold mix	208(25.43%)	111(25.81%)	0.88
Grass mix	22(2.69%)	9(2.09%)	0.52
Tree pollen mix	50(6.11%)	23(5.34%)	0.59
Egg	173(21.15%)	110(25.58%)	0.08
Cow’s milk	258(31.54%)	119(27.67%)	0.16
Shrimp	82(10.02%)	30(6.98%)	0.07
Beef	118(14.43%)	44(10.23%)	0.04
Shellfish	25(3.06%)	20(4.65%)	0.15
Crab	117(14.30%)	39(9.07%)	0.01
Mango	54(6.60%)	33 (7.67%)	0.48
Cashew nut	242(29.58%)	107 (24.88%)	0.08
Pineapple	18(2.20%)	6 (1.39%)	0.33

All children were divided into three groups based on age (1–3, 4–7, and 8–14 year). The sensitization to total inhaled and food allergens was significantly different among age groups (*P* < 0.01) (Table 4). The positivity rate of inhaled allergens increased with age, while conversely, the positivity rate of food allergens decreased with age. The positivity rate of inhaled allergens in children aged 1–3 year was lower than that of food allergens (*P* < 0.01). The opposite trend was observed in children aged 8–14 year (*P* < 0.01). The inhaled allergen sIgE positivity rates of *D. pteronyssinus*, house dust, cat dander, amaranth, and mixed mold were low in the 1–3 year group and increased with age, while the positivity rates of egg, cow’s milk, crab, and cashew showed the opposite trend (Table 5).

**Table 4:** Allergen sensitization in different age groups

Allergens	Number of sIgE-positive patients (%)			*P* value
	1–3 yr	4–7 yr	8–14 yr	
Number of patients	81	501	666	
Inhaled	49 (60.49%)	357 (71.26%)	593 (89.04%)	< 0.001
Food	68 (83.95%)	366 (73.05%)	392 (58.86%)	< 0.001

**Table 5:** The positivity rate of sIgE in different age groups

Allergens	Number of sIgE-positive patients (%)			*P* value	
	1–3 yr	4–7 yr	8–14 yr		
	*n* = 81	*n* = 501	*n* = 666		
*D. pteronyssinus*	23 (28.40%)	284 (56.69%)	509 (76.43%)	< 0.001	
House dust	10 (12.35%)	79 (15.77%)	169 (25.38%)		< 0.001
Mulberry	0 (0%)	16 (3.19%)	17 (2.55%)	0.25	
Cat dander	24 (29.63%)	61 (12.18%)	56 (8.41%)	< 0.001	
Dog hair	17 (20.99%)	74 (14.77%)	83 (12.46%)	0.09	
Cockroach	2 (2.47%)	13 (2.59%)	32 (4.80%)	0.11	
Amaranth	0(0%)	24(4.79%)	56(8.41%)	0.002	
Mold mix	11(13.58%)	156(31.14%)	152(22.82%)	< 0.001	
Grass mix	1(1.23%)	10 (2.00%)	20 (3.00%)	0.42	
Tree pollen mix	4 (4.94%)	33 (6.59%)	36 (5.41%)	0.65	
Egg	34 (41.98%)	136 (27.15%)	113 (16.97%)	< 0.001	
Cow’s milk	42 (51.85%)	173 (34.53%)	162 (24.32%)	< 0.001	
Shrimp	12 (14.81%)	43 (8.58%)	57 (8.56%)	0.16	
Beef	12 (14.81%)	67 (13.37%)	83 (12.46%)	0.79	
Shellfish	6 (7.41%)	20 (3.99%)	19 (2.85%)	0.10	
Crab	19 (23.46%)	68 (13.57%)	69 (6.46%)	0.002	
Mango	6 (7.41%)	38 (7.58%)	43 (6.46%)	0.75	
Cashew nut	39 (48.15%)	168 (33.53%)	143 (21.47%)	< 0.001	
Pineapple	1 (1.23%)	9 (1.80%)	14 (2.10%)	0.84	

### Characteristics of the total serum IgE level distribution

As shown in Table 6, there were 164 children with total IgE < 100 IU/ml, including 96 boys and 68 girls, with the following age distribution: 1–3 year (*n* = 16); 4–7 year (*n* = 92); and 8–14 year (*n* = 56). Total IgE ranged from 100 to 200 IU/ml in 79 patients, including 49 boys and 30 girls, as follows: 1–3 year (*n* = 8*)*; 4–7 year (*n* = 30); and 8–14 year (*n* = 41). A total of 1005 patients were classified in the high-concentration group, including 673 boys and 332 girls: 1–3 year (*n* = 57); 4–7 year (*n* = 379); and 8–14 year (*n* = 569). There was no significant sex difference in total IgE level (*P* > 0.05), but there was a significant age difference (*P* < 0.01) and the total IgE level increased with age.

**Table 6:** Total serum IgE level in different age groups

Total IgE	< 100 IU∕ml	100–200 IU∕ml	> 200 IU∕ml	
1–3 yr	16	8	57	81
4–7 yr	92	30	379	501
8–14 yr	56	41	569	666
	164	79	1005	1248

## Discussion

Allergic rhinitis is one of the most common allergic diseases in children, which not only leads to a decline in quality of life but also affects school performance [2, 11]. The prevalence of AR has increased significantly worldwide, with differing regional main types of allergens and prevalence. A cross-sectional study showed an AR prevalence of 11.7–21.2% in Europe [12] and the worldwide AR prevalence is expected to increase further over the coming decades. A survey conducted in 11 major cities in China showed that the self-reported prevalence of AR was about 8.7–24.1% [13].

The etiology of AR is still unclear and its pathogenesis is likely related to the combined influences of both genetic and environmental factors [3, 14]. Allergen sensitization is one factor that plays a key role in the development of AR and has an important impact on prognosis for children with AR [15]. Allergen avoidance is the primary strategy for AR treatment [16]. Strategies for reducing the generation of allergen-related sIgE include changing and controlling the living environment, reducing allergen contact during exposure, and avoiding allergen exposure, especially in infants. Such measures may even lead to complete remission of the allergen sensitization state. Several studies have shown that reducing allergen exposure can reduce clinical symptoms and improve quality of life in patients with perennial AR, as well as nasal and eye-related symptoms in patients with allergic nasal conjunctivitis [17, 18]. However, allergens are affected by multiple factors such as regional environment, climate, temperature, and humidity [19]. Consequently, different AR triggers are found in different geographic and situational locales. Therefore, clarifying the characteristics of allergen distribution in this region of China is important for the prevention and treatment of AR.

The results of this study showed the most common inhaled allergen in children with AR in Changzhou was *D. pteronyssinus* and the positive detection rate of inhalation allergens is significantly higher than that of food allergens, which is similar to results of several previous studies [20–22]. The second-ranked inhaled allergen was a mold mix of spot penicillin, crossstrepsporum, mycospore, and *Aspergillus fumigatus*. This finding may be related to Changzhou’s location in a subtropical monsoon climate area conducive to the breeding of mites and mold, as well as the centralized treatment of local allergen-positive children. At the same time, the positive rate of dog hair and cat dander in this study was also high, likely because Changzhou is located in an economically developed area of China, with high living standards and more pet breeding.

Some studies have demonstrated that allergic diseases are more common in females than in males [23, 24], while other studies have reported higher allergen sensitization rates in men than in women [25, 26]. We found no sex differences in the positivity rates of inhaled and food allergens, which is consistent with the findings in some previous studies [27, 28]. The reason for the difference remains unclear and further studies should be conducted to explain any observed sex-related differences. The sIgE positivity rates of mulberry, cat dander, dog hair, amaranth, beef, and crab were higher among males than females. The reason for this difference may be related to different exposure opportunities to different allergens and differences in immune function between males and females. We also found that the positivity rates of overall inhaled allergens in boys and girls were significantly higher than that of food allergens, which may be related to the fact that most of the patients in our center have AR.

The characteristics of allergen sensitization in children with AR of different ages vary. Several epidemiological studies on allergic diseases showed that infants < 2 year mainly tested positive for food allergens, while school-aged children mainly tested positive for inhalation allergens [29–31]. Consistent with this trend, the overall positivity rate of inhaled allergens in the 1–3 year group was lower than that of food allergens, while the highest percentage of children sensitized to inhaled allergens was observed in the 8–14 year group. At the same time, the inhaled allergen sIgE positive rates including *D. pteronyssinus*, house dust, cat dander, amaranth, and mixed mold were low in the 1–3 year group and increased with age, while the positivity rates of egg, cow’s milk, crab, and cashew showed the opposite trend. We also found that total serum IgE levels increased with age, suggesting that the allergic status in children with AR may be progressive without the implementation of effective allergen avoidance and control.

Notably, this study had some limitations. First, children had numerous daily exposure to allergens, but only the positive rate of sIgE was analyzed in this study. Second, the present study was retrospective in nature and may have a bias associated with patient selection. Finally, the severity of AR cannot be assessed by allergen distribution. Allergens are greatly affected by geographical and environmental factors, and this study only reflected the allergen distribution of children with AR in this region. Strategies to improve the rigor of future studies include expansion of the sample size and grouping of patients by disease severity to explore whether severity correlates with the positivity rate of allergens.

## Conclusion

The prevalence and distribution of allergens in pediatric patients with AR in Changzhou exhibited differences in sex and age, and *Dermatophagoides pteronyssinus* is the most common allergen in Changzhou. These findings may be helpful to provide a basis for epidemiological research in children with allergic rhinitis in this region and guide more evidence-based and individualized prevention and treatment of this disease. The findings of this study may also be applicable to patients in other regions with similar climatic conditions and lifestyles in eastern China.

## Data Availability

The datasets used and/or analysed during the current study available from the corresponding author on reasonable request.
